# Sporting Habits of Urban Runners: Classification According to Their Motivation

**DOI:** 10.3390/ijerph16244990

**Published:** 2019-12-08

**Authors:** David Parra-Camacho, María Huertas González-Serrano, Rómulo Jacobo González-García, Ferran Calabuig Moreno

**Affiliations:** 1 Department of Physical Education and Sports, Faculty of Physical Activity and Sport Sciences, Universitat de València, 46010 Valencia, Spain; david.parra-camacho@uv.es; 2 Department of Teaching and Learning of Physical Education, Plastic and Music Education, Universidad Católica de Valencia, 46110 Valencia, Spain; m.huertas.gonzalez@ucv.es; 3 Universidad Internacional de Valencia, 46002 Valencia, Spain; romulojacobo.gonzalez@campusviu.es

**Keywords:** runners, typology, sports, clusters, motives, citizens’ sports habits, urban runners

## Abstract

This study analyses the sporting habits of runners participating in short-distance urban running events to identify groups with different motivations towards the practice of endurance running and participation in urban running events. A sample consisting of 937 participants in the Valencia running circuit was interviewed using a questionnaire consisting of a scale of 22 items to analyse their motives for participating in popular races. An exploratory and confirmatory factorial analysis was carried out to check the validity of the instrument, and the analysis identified the following four factors into which the indicators were grouped: psychological and physical motives (3 items), social motives and interest in running events (5 items), occupation of time and social recognition (6 items) and competitive and material motives (3 items). Subsequently, a cluster analysis was performed by combining hierarchical and non-hierarchical methods, and the analysis identified the following three groups of runners with different characteristics: individual hedonists (n = 276), enthusiasts (n = 312) and socializing hedonists (n = 349). Enthusiastic runners consider most motives important when participating in running events, individual hedonists consider individual psychological and physical motives important, and socializing hedonists consider personal and social motives and interest in sport important. Variables related to age, educational level, annual income level, frequency of running, how the individuals went out to run and the level of the runners contributed to differentiating the identified groups. The results confirm the heterogeneous nature of urban runners.

## 1. Introduction

There is a rising interest in running as an activity; it is one of the most popular forms of sports participation in Western Europe, with approximately 50 million participants, according to Breedveld et al. [1]. Additionally, according to data from the latest Survey of Sports Habits in Spain, among the people who practice some type of physical activity or sport, the percentage of the population who practice running is 30.4% [2]. The practice of running has increased in popularity by 6.6% between 2010 and 2015 (from 23% to 30.4%, respectively), and it is now the third most-practised sport. Along the same lines, the number of urban races has increased considerably in recent years. According to the Municipal Sports Foundation of Valencia, the number of popular races held during 2017 in the city was 39 [3].

Since the 1960s, the sport of running has been growing internationally as part of a specific trend which is defined as the “desportification and deinstitutionalization of the sport sector” [4]. This trend implies the practice of sport is performed from a healthy, relaxing, recreational or pleasurable point of view, rather than a competitive one [5,6]. People often want to participate in sporting events to become amateur athletes and take on the challenges of a sporting and healthy lifestyle, seeking the strong emotions provided by sporting rivalry, as well as establishing social relationships and eliminating anxiety, depression and mood changes with the aim of improving the quality of life [4].

Running generates numerous benefits to improve health and prevent the problems associated with a sedentary lifestyle. Running is an activity characterized by a health-oriented approach, popularity and accessibility, because it has few economic conditions or specific infrastructures for its practice and can be practised anywhere, at any time [7,8]. Although this activity attracts a large number of practitioners of all ages, there are problems associated with its abandonment, due to injuries, motivational deficiencies and even negative addictions. In this sense, some works, such as those of Zarauz and Ruiz-Juan [9] and Ruiz-Juan and Zarauz [10], have analysed the negative addiction to running in Spanish runners and have identified some of the variables that determine this addiction.

The motivations of runners to participate in endurance races have been widely analysed in multiple countries, specifically the international contributions of Carmack and Martens [11], Clough et al. [12], Thornton and Scott [13] and Ogles, Masters, and Richardson [14]. These studies have identified the reasons why people choose to run, and they highlighted well-being, physical health, physical condition, psychological health, status, goal achievement, tangible rewards, social influences, availability, and other reasons. Similarly, Masters, Ogles and Jolton [15] designed an instrument to evaluate the motivation of marathon runners, namely, the Motivations of Marathoners Scales (MOMS), which contains 56 reasons to run that are grouped into 4 general categories (psychological, achievement, social and physical health).

On the other hand, in the Spanish context, Llopis and Llopis [16] studied a group of runners by taking into account variables such as gender, age, level of studies, number of years running, number of years participating in races, affiliation with an athletic club and having a trainer. In this study, the main reasons highlighted by runners for their practising running were the satisfaction produced by running, the achievement of a personal goal, physical fitness, social interaction and an interest in the sport. This study also indicated the influence that being a member of an athletic club and being supervised by a sports trainer has on one’s reasons for running.

Most papers have focused on the study of the motivations of marathon runners [17,18,19,20,21] and, to a lesser extent, shorter distance runners [14,16,22,23,24]. Most of these studies have observed that both sociodemographic variables and those related to training habits influence the reasons why runners participate in marathons [20].

However, there are few studies that identify clusters or groups with different characteristics depending on the motivation for running among urban runners. In this sense, the work of Ogles and Martens [18] should be highlighted, although the study is analysed from the point of view of marathon runners. These authors identified the following five groups with different characteristics according to sociodemographic, training and performance variables: running enthusiasts, lifestyle managers, personal goal achievers, personal accomplishers and competitive achievers. The authors observed that personal reasons were more determinant than social or competitive reasons in all identified groups.

The work of Rohm et al. [25] identified the following four clusters with different motivations among U.S. runners: healthy joggers, social competitors, actualized athletes and devotees. In this work, the authors observed that the variables related to training, experience as a runner, age and gender contributed to significantly differentiating the identified clusters. A study by Vos et al. [26] interviewed Belgian runners and identified four groups with different motives towards the practice of running: traditional/generic runners (35.2%), social competitive runners (14.5%), individual fitness runners (12.2%), individual competitive runners (24.9%) and companionship runners (13.2%). These authors found that variables such as age, average distance completed, participation in popular racing events in the last 12 months and expenditure on sports equipment contributed significantly to differences in the groups found.

From a qualitative point of view, the study on the sociocultural typology of urban runners carried out by Llopis and Llopis [23] should be highlighted. This work identified the following four groups of runners with different characteristics according to the relational character of the practice (individual vs. group) and the degree of technical formalization of the running practice: hedonistic solitaire, competitive individualists, relational runners and disciplined groupists. Hedonistic solitaire runners are characterized by being urban runners with both a scarce technical formalization and a weak relational character; competitive individualists are characterized by being runners with low levels of relational dimension but high levels of technical formalization. Relational racers are characterized by a strong relational character but a minimal degree of formalization of running. Finally, the individualized groupists are the runners who present high levels of both dimensions of technical formalization and relational character.

Studies that consider the population of urban runners from a heterogeneous point of view and that make it possible to identify clusters with different reasons or motives towards the practice of continuous running are not frequent. For this reason, the main objective of this work is to identify groups of urban runners according to their motivation to practice this type of physical activity and to participate in popular races within the running circuit of Valencia (Spain). This circuit is characterized by being made up of ten urban races, each with a distance of between 5 and 10 km. Additionally, the sociodemographic characteristics of the identified clusters and the sporting habits that define each group are analysed.

## 2. Materials and Methods

### 2.1. Sample, Procedures and Questionnaire

A quantitative cross-sectional study was carried out among the participants in the running circuit of Valencia once the last race of the circuit was finished. The sample was collected by using intentional and non-probability sampling. A sample consisting of 937 participants in the popular race circuit of the city was collected during 2014. The survey collection process was carried out between 15 December 2014 and 15 January 2015, once the last race of the circuit was finished, by using a three-part online questionnaire.

The first part collected sociodemographic data from questions referring to the age, sex, occupation, educational level and income level of the interviewees.

The second part examined the sports habits of the participants, with questions referring to the number of years they have been running, the frequency with which they run, their preferred distance when participating in a popular race, their membership in a club, whether they are federated, with whom they usually run, how far they usually run per week, the athletic level they consider themselves to be at and the number of long-distance races they have completed (half marathons and marathons).

The third part examined the motivations as to why the participants usually participate in popular races. The items proposed by Barrios and Cardoso [27] were used to measure the perceptions of the participant’s motivations as to why they usually participate in popular races. This Likert-type scale had five response options: 1 = not important, 2 = not very important, 3 = important, 4 = very important and 5 = extremely important. This scale consisted of 23 items; however, one indicator was dropped (being a part of my preparation for national defense) to avoid possible confusion because it does not fit the reality of the Spanish social context.

The survey collection procedure was delivered via the Internet through an online survey. It was decided to use the Internet to collect surveys from the sportsmen and women due to the difficulty of collecting a large sample of participants during the various races in the circuit, as many of the runners participated in the same events or recovered from the same effort.

The sociodemographic characteristics of the sample are summarized in Table 1; the participants had an average age of 39.40 (SD = 9.38), with ages between 18 and 71. According to gender, 75.5% of the participants were men, and 25.5% were women; the majority were employees (76.8%) who had attained a university education (58.1%) and had an income level of less than 18,000 euros per year (50.3%).

### 2.2. Statistical Analysis

The statistical treatment was performed using the SPSS, FACTOR and EQS programmes. First, the total sample was divided into two samples. To check the validity of the scale, an exploratory factorial analysis (EFA) was performed using the first sample (n = 465), and a confirmatory factorial analysis (CFA) was performed with the second sample (n = 472). The EFA was performed following the recommendations of Lloret-Segura et al. [28] and used the maximum likelihood (ML) method and oblimin direct rotation. The optimal implementation of the parallel analysis procedure was used to determine the number of factors [29]. The fit of the model was observed by means of the coefficients of the root mean square of the residuals (RMSR), as well as the gamma index or the goodness-of-fit (GFI) proposed by Tanaka and Huba [30]. The RMSR should be less than 0.05 [31], and the GFI value should be less than 0.95 [32]. Another indicator that was taken into account was the generalized H index (GH) (>0.80), which was used to analyse the replicability of the factors derived from the EFA [33]. The measures for the sample adequation of Kaiser-Meyer-Olkin (KMO) were also observed, as was Bartlett’s sphericity test. Additionally, the items with factorial loads below 0.30 or above this value in two or more factors were eliminated before carrying out the next EFA. Finally, the theoretical interpretability of the factorial solution extracted from the EFA was tested. The interpretability of the factorial solution was made from the dimensions of the factors on the motives for which runners participate in endurance races that have been identified in previous studies [18,20].

After the EFA was applied, an CFA was performed on the factorial solution derived from the EFA, using the robust maximum likelihood estimation (MLR) with the aim of correcting the possible absence of multivariant normality by using statistics such as the χ^2^ of Satorra-Bentler [34]. Thus, for the evaluation of global fit, different goodness-of-fit indexes recommended in the literature were used [35], such as the signification of the chi-squared test and its robust correction offered by Satorra-Bentler (S-B χ²) [36]. In addition, other coefficients were calculated that allowed for testing the adequation of the proposed models, such as the ratio of χ² and its degrees of freedom (χ^2^/df; [37]), with acceptable values being less than five [38]. The χ^2^/df is an indicator recommended by the literature to check the fit of the model. Normally, the use of three or four adjustment indices is recommended to provide adequate evidence of the fit of the model, requiring the reporting of the value χ^2^ and the associated degrees of freedom [39]. In the same way, the coefficients of the indexes of the robust goodness-of-fit of the proposed model, the compared fit index (CFI) and the incremental fit index (IFI) were tested. For these indicators, a good fit was considered as values above 0.90 [40]. Finally, the root mean square error of approximation (RMSEA) was shown, with a score below 0.08 being considered a good fit [41].

In the evaluation of the reliability of the scale, three measurements were taken into account: Cronbach’s alpha, the composite reliability (CR) and the average variance extracted (AVE) for each factor [39]. Additionally, the convergent validity was tested through the significance of the factorial loads in their respective dimensions and the values of the associated *t* tests. Furthermore, the discriminant validity, which has to do with seeing the clear distinction between any pair of constructs, was evaluated using the method suggested by Fornell and Larcker [42]. This method demonstrates discriminant validity if the square root of the AVE value of a determined factor is greater than the correlation coefficients between the determined factor and any other factor in the proposed scale. The other criteria for assuring discriminant validity indicate that the correlations between the different pairs of factors must be less than 0.85 [35].

Finally, to identify groups of participants with different characteristics according to the reasons for which they usually run, a cluster analysis was carried out using the statistical program SPSS, version 24, for Windows (IBM, Armonk, NY, USA), with the items derived from the CFA. Two methods of the estimation (hierarchical and non-hierarchical) of the cluster solution were combined to optimize the results. The hierarchical cluster analysis was performed using Ward’s grouping process method and the Euclidean distance squared as a similarity measure. From the groups proposed in the previous analysis, a non-hierarchical analysis was applied using the K-medians method, which used as its initial centres the means of the variables obtained for each cluster solution of the hierarchical analysis. To define the characteristics of the group profiles and to evaluate their predictive validity, ANOVAS and chi-square tests were performed with variables that were not included in the initial analysis. The value of the contingency coefficient (C) was also used to check the intensity of the association or the size of the effect of the related variables.

## 3. Results

### 3.1. Validation of Measurement Scale

#### 3.1.1. Descriptive Statistics

To analyse the validity of the scale of reasons as to why runners usually take part in popular races, the mean, standard deviation, asymmetry and kurtosis of each indicator were first analysed (see Table 2). The reasons that runners consider very important (i.e., they have values close to the value of 4 on the Likert scale) are those that refer to "feeling pleasure in running” (M = 4.01; SD = 0.88), “fulfilling the goal I set for myself” (M = 3.90; SD = 0.91), “checking my physical condition” (M = 3.76; SD = 0.84), “having fun during competition” (M = 4.14; SD = 0.84), “being attracted to the sport” (M = 3.93; SD = 0.86), “being proud of myself” (M = 4.12; SD = 0.90) and “feeling more confident” (M = 3.70; SD = 1.12). On the other hand, the reasons that the runners consider as being a little or not important are those that refer to “the prestige of this competition” (M = 2.33; SD = 1.14), “others feeling proud of me” (M = 2.40; SD = 1.20), “being a part of my preparation for another sport” (M = 2.38; SD = 1.25), “others seeing me competing with my friends and family” (M = 2.08; SD = 1.09), “beating other teammates” (M = 1.91; SD = 1.05), “wanting to be selected to represent my country” (M = 1.40; SD = 0.84), “getting a better time than another teammate” (M = 1.80; SD =1.06) and “wanting to get some material stimulus (T-shirt, runner’s bag, etc.)” (M = 2.18; SD = 1.21). The values of asymmetry and kurtosis were acceptable in most variables, as they were lower than 3.0 [43]. However, it was decided to eliminate item M20 from the factorial analyses because it had a high kurtosis value (K = 5.70).

#### 3.1.2. Exploratory Factor Analysis

An EFA was conducted for the 21 items that measured the motivations of runners participating in popular races using half of the sample (n = 465) (see Table 3). The parallel analysis procedure suggested grouping the indicators into two factors. However, to achieve a better theoretical interpretation, it was decided to perform the EFA with four factors. To comply with the above criteria, two items with factorial loads lower than 0.30 or higher than 0.30 in two or more factors were eliminated: M4 and M20.

The indicators were grouped into the following factors: psychological and physical motives (4 items), social motives and interest in running events (6 items), occupation of time and social recognition (6 items) and competitive and material motives (3 items).

To check the fit of the model, the RMSR and gamma or GFI index coefficients were analysed, which showed the values within the recommended cut-off points: RMSR = 0.04 (<0.05) GFI = 0.99 (>0.95). Additionally, the generalized H index showed values higher than 0.80 in all the factors detected by the EFA (ranging between 0.80 and 0.91), indicating a good replicability of the dimensions in other studies. The variance explained by the 19 items grouped in the four factors was 60.92%.

#### 3.1.3. Confirmatory Factorial Analysis

After the EFA was applied, a CFA was conducted with the second part of the sample (n = 472) using the factorial solution proposed by the EFA. This solution did not show a good fit, and it was necessary to re-specify the model by eliminating two indicators that presented high residual values and reduced factorial loads: M1 and M2.

For this final model (17 items in four factors), we verified that the fit was adequate (χ ^2^ = 444.36, df = 113, *p* < 0.01; χ^2^/df = 3.66), as indicated by the literature [44], taking into account that χ^2^ is very sensitive to sample size (n = 472) and that with large samples [35,45], chi-square values increase, which could erroneously indicate a poor fit of the model data [46]. The rest of the goodness-of-fit indexes showed a good fit ((RMSEA = 0.078 (IC = 0.072–0.085); CFI = 0.91; IFI = 0.91)).

The Cronbach’s alpha, CR and AVE measurements were used to analyse reliability (see Table 4). The Cronbach’s alpha values were higher than the 0.70 value recommended by the literature [39]. This criterion was also met in the case of the CR values, ranging from 0.80 to 0.85. Finally, for the AVE values, factors 1 and 4 were found to have values greater than the 0.50 value recommended by the literature [47]. However, factors 2 (0.46) and 3 (0.49) had values lower than 0.50. According to Hatcher [48], when the reliability of the construct is acceptable, a marginally low AVE value can be accepted (see Table 4). It was therefore decided to retain these factors without combining them with other factors because of their relevance and theoretical interpretability.

To analyse the convergent validity, it was verified that the values of the t tests associated with the factorial loads of the items were higher than 1.96 (*p* < 0.05), specifically ranging from 5.86 to 17.99. Regarding the discriminant validity, we found that all the correlations between the various factors were lower than 0.85, thus fulfilling this criterion (see Table 5). Also, it was verified that the square root of the AVE was superior to the correlation between pairs of factors; thus, this criterion was also fulfilled (see Table 5).

### 3.2. Identification and Description of Clusters

After analysing the validity and reliability of the scale of motives as to why runners usually participate in popular races, a cluster analysis was carried out to identify the groups with different characteristics depending on the motives for why they participate in popular races.

Following the procedure recommended by Hair et al. [39], a hierarchical cluster analysis was first performed using Ward’s method of observing the increase in the agglomeration coefficients between clusters two and three, three and four, and four and five. After observing these coefficients, the solutions of two, three, four and five groups were used to apply the second analysis of the k-median clusters by using the initial centres from the hierarchical cluster analysis. These solutions have been analysed in previous work in this area [18,25,26]. It was considered appropriate to contrast all the solutions because there are few previous studies that identify clusters of urban runners, as mentioned in the theoretical framework.

The choice of a suitable cluster solution depends on the theoretical foundations, common sense and practical judgement of the researcher [39]. In this case, it was decided to adopt the solution of three clusters, since this choice facilitated the interpretation and identification of the groups of runners, thereby allowing for the differentiation and identification of three clear groups of runners with different motives about their participation in popular races.

Table 6 shows the mean value (centroid) for each of the 17 motivational variables included in the analysis and the results of an ANOVA test carried out to confirm the significant difference between the groups (statistical F and significance). As we can see in this table, the variables that most distinguish the clusters correspond to the occupation of time and social recognition factors: “the prestige of this competition” (F = 468.57) and “to demonstrate my interest in the sport” (F = 415.06). The items that least differentiate the clusters are “having fun during the competition” (F = 47.61) and “checking my physical condition” (F = 69.59).

Cluster 1 was labelled the “individual hedonists” (n = 276; 29.45%) because they only consider important aspects of individual character at the psychological level (“feeling proud of myself” or “feeling more confident in myself”) and the physical level (“checking my physical condition”), although they also point out very important aspects related to personal fun during the races or an attraction to the sport. This group does not consider the occupation of leisure time or social recognition as important reasons for participating in popular races (M = 1.62) nor does it consider competition-related motives or materials to be relevant (M = 1.51).

Cluster 2 was identified as the “enthusiasts” (n = 312; 33.30%) because it is the group that considers all the reasons as important in determining their participation in popular races, highlighting both personal (M = 4.32) and social reasons and interest in the sport (M = 4.12). These participants also consider as important the possibilities offered by popular races to occupy their free time and make their life more meaningful, the social recognition involved and the usefulness of running in their preparation for other sports. These participants attach less importance to the competitive and material aspects (M = 2.76).

Cluster 3 was labelled the "socializing hedonists" (n = 349; 37.25%) because they value not only personal motives but also those of a social nature and an interest in the sport as being important. Thus, in this group, high scores are observed in the factors related to personal psychological and physical motives (M = 3.99) and in social motives and interest in running events (M = 3.65). These participants consider the motives related to social recognition and competitive and material reasons to be less relevant (M = 1.61) than other motives.

### 3.3. Profile of the Groups

The profile of the respondents that comprised each cluster was obtained from other independent variables that, in turn, allowed us to ensure the predictive validity of the groups. Table 7 shows the percentages for each sociodemographic variable and the related sports habits according to each cluster.

The sociodemographic variables in which statistically significant differences were detected were those related to age (F(2, 934) = 4.07; *p* < 0.05), educational level (χ^2^(6) = 31.31; *p* < 0.001) and annual income level (χ^2^(10) = 21.73; *p* < 0.05). The size of the effect of the contingency coefficient in these variables was reduced and ranged from 0.09 to 0.18 for the different variables (see Table 7). Regarding the variables related to sports habits, it was found that the frequency with which the participants went out to run during the week (χ^2^(6) = 14.79; *p* < 0.05), how they went out to run (χ^2^(2) = 7.33; *p* < 0.05) and the level at which they saw themselves as a runner (χ^2^(4) = 26.31; *p* < 0.05) contributed to significantly differentiating the identified groups.

The group of “individual hedonists” is characterized by a mean age of 39.52 (SD = 8.80) with a majority percentage of men (79.35%), employees (80.80%), a university education (67.75%) and a higher level of annual income (54.7% over 18,000 euros) when compared to the other groups. According to their sporting habits, almost half of the members of this group run three to five times a week (48.19%). Their preferred distance in popular races is either less than 7.5 km (41.67%) or 7.5 to 10 km (23.19%), with a significantly higher proportion of the subjects indicating a preference for shorter distance races compared to the group of “socializing hedonists”. The majority of this group tend to run alone (65.22%), presenting a significantly higher proportion of solo runners if we compare the result mainly with the group of “enthusiasts”. The participants in this group mainly consider their level as a runner to be medium (50.36%) or low (45.29%), with significantly lower and higher proportions of these levels, respectively, than in the group of “enthusiasts”. The average distance in kilometres that they run per week is 26.93 (SD = 16.91), the average number of years that they have run is 7.35 (SD = 7.76), and the mean number of long-distance races they have participated is 4.51 (SD = 10.79) for half marathons and 0.89 (SD = 2.84) for marathons.

The group of ‘‘enthusiasts’’ has the youngest mean age (M = 38.25; SD = 9.85) and a majority percentage of men (75.96%) and employees (73.92%). Although a large portion of this group has a university education (48.72%), it is the group with the lowest proportion of runners with this level of education if we compare it with the other two groups. It is also the group with the highest proportion of subjects with the lowest level of annual income (58.98% with less than 18,000 euros). From the point of view of its sporting habits, it is the group that presents a greater proportion of subjects who run more frequently (54.81% indicate that they run three to five times a week) when compared with the other two groups. The preferred distance in popular races in this group is either less than 7.5 km (34.29%) or 7.5 to 10 km (30.45%), and the members usually run alone (54.49%); however, this proportion of respondents is significantly lower when compared to the group of “solitary hedonists”. These members consider their level as a runner to be medium (66.67%), with a significantly higher proportion of medium runners than the group of “enthusiasts”. This group is also the group with the highest proportion of runners associated with any sports club (45.19%) when compared to the other groups. The average distance in kilometres that they run per week is 29.04 (SD = 16.08), the average number of years that they have run is 6.91 (SD = 7.85) and the mean number of long-distance races they have participated in is 5.68 (SD = 21.22) for half marathons and 0.78 (SD = 2.25) for marathons.

Finally, the group of “socializing hedonists” has the highest mean age (M = 40.32; SD = 9.56) and a majority proportion of men (71.92%), although this group has the lowest proportion of men when compared with the other groups. This group also has a majority percentage of employees (76.50%) and those with a university education (58.74%). Most members have an annual income level of less than 24,000 euros (70.20%). From the point of view of their sporting habits, this group presents a majority proportion of runners who go out with a frequency of three to five times a week (53.87%). Their preferred distance in popular races is either less than 7.5 km (31.52%) or 7.5 to 10 km (31.52%), and they usually run alone (61.32%). They consider their level as a runner to be medium (52.72%) but with a significantly lower proportion of medium runners than the “enthusiasts” group. Both the average distance in kilometres that they run per week (M = 26.97; SD = 14.71) and the years that they have been running (M = 7.28; SD = 8.57) are similar to those of the “individual hedonists” group. The mean number of their long-distance races was 5.11 (SD = 14.95) for half marathons and 0.84 (SD = 2.74) for marathons.

## 4. Discussion

This paper analyses why runners participate in popular races by identifying groups of runners with different motives. After analysing the validity and reliability of the scale of motives as to why runners participate in popular races, four factors related to personal motives at the psychological and social levels, social motives and interest in running events, the occupation of time and social recognition, and competitive and material motives were identified. Although runners are generally considered as global in some media, runners cannot be viewed as a homogeneous group as they constitute a socially heterogeneous universe within which various subcultures can be identified [23].

Previous studies have used scales that have measured the perception of motives in populations of runners who participate in endurance races such as marathons [18,49]. In this study, we focus on validating a scale in runners who participate in shorter urban endurance races. The factors identified coincide with those observed in previous works that have validated instruments for analysing motives in marathon runners [18,49].

Additionally, in this paper, we identify three groups with different motives surrounding their participation in popular races: “individual hedonists”, “enthusiasts” and “socializing hedonists”. The delineation of these groups was made using the evaluations of importance that the respondents gave for the different motives and the tendencies observed in previous works on the segmentation of runners according to their participation motives [18,23,25].

Variables such as age [18,25,26], educational level and income level were found to contribute to differentiating the identified groups. Additionally, some variables related to the participants’ sports habits, such as the frequency with which they go out to run [18,25], their company when going out to run [18,25], or the level that they consider themselves to be at as a runner, contributed to differentiating the identified clusters. However, in this study, it was not observed that the variables related to the volume of kilometres run during the week, the number of years of running or the preferred distance in the races contributed to differentiating the groups. In other studies, it has been observed that some of these variables related to sports habits significantly differentiated the study groups [25,26]. Therefore, the need to look at runners from a heterogeneous point of view has been observed in other studies [18,25,26].

The identified groups present some characteristics similar to those observed in previous works. In the case of the cluster of “enthusiasts”, it can be observed how most of the motives (psychological, physical, social and social recognition) are considered as important when participating in popular races. This group has been detected in the work of Olsen et al., [18], from which we extracted the name “running enthusiasts”, and shares characteristics with the “social competitors” and “actualized athletes” group identified in Rohm’s study [25]. However, from the point of view of sociodemographic characteristics, the three groups were very similar in age to each other, but the “enthusiasts” were the youngest group, and in the work of Olsen et al., [18], it was the oldest. In this study, the group with the smallest proportion of people who go out to run alone had a lower average number of years of running experience and a greater proportion of people involved in a sport club when compared with the other groups. In line with the work of Olsen et al., [18], this “enthusiasts” group was more likely to run with others.

On the other hand, the group of "solitary hedonists" is characterized by giving importance to individual psychological and physical motives but giving little importance to aspects related to social recognition, occupation of free time, competition or material aspects. This group was labelled the “solitary hedonists” because of the members’ interest in individual pleasure rather than in other aspects of social interaction. The name of this group was also identified in the Llopis and Llopis study [23] as the name of runners who run individually and with few external social or training conditions. This group also shares characteristics with the group of “healthy joggers” identified in Rohm’s study [25]. In this sense, the group stands out for running less frequently every week, running fewer kilometres every week, running alone, averaging more years of running experience running and preferring to run with short distances (less than 10 km) in greater proportions than the other groups. From a sociodemographic point of view, it is the group with the highest proportion of men, those with a university education, employees and a higher level of annual income when compared to other groups.

Finally, the group of “socializing hedonists” is characterized by giving importance not only to personal motives but also those related to the social nature of and interest in the sport; this group discards as relevant the motives related to external social recognition and those of a competitive or material type. Additionally, this group follows individual pleasure through both personal achievements and through the possibilities of social interaction offered by participation in popular races. This group shares characteristics with the group of "relational runners“ identified by Llopis and Llopis [23] and the group of "social competitors” in Rohm’s work [25]. According to their sporting habits, the “socializing hedonists” are characterized by running at a frequency of three to five times a week in greater proportion than the other groups, by preferring to run alone and preferring short distances in popular races. The percentage of the group’s members associated with a sports club is slightly lower than that of the “enthusiasts”, and the average number of years they have been running is slightly higher than that of the “enthusiasts”. This is the group with the highest average age, with the highest proportion of women compared to others and with a more balanced distribution among its members in terms of annual income level.

### 4.1. Practical Implications

This study corroborates the existence of groups with different motives for their involvement in popular races in the Spanish context. It makes it possible to identify the sociodemographic profile of each group, as well as their sporting habits and their behaviour patterns. Such studies on urban runners allow administrations to understand runners’ motivations and interests, with the aim of designing sports policies that allow administrations to attend to the interests of these groups, which usually represent an important portion of the people who practice some type of physical activity and sport. From the results, it can be deduced that the majority of the urban runners interviewed are individual practitioners with an interest in short rather than long-distance races, a weekly training frequency of 3 to 5 times, a running volume of approximately 27–28 kilometres per week, a self-perceived intermediate running level and a running experience of less than ten years.

Although the city of Valencia has numerous and adequate spaces for the practice of this sport, a healthy and responsible practice should be encouraged by offering advice on public sports services with the aim of prolonging adherence to sports practice and contributing to improving the quality of life of the population. In this way it also contributes to being a socially responsible event [50]. Finally, it is recommended that the organisation of popular running events with short or medium distances, that allow the massive participation of the local population, should continue to be supported.

### 4.2. Limitations and Future Lines of Research

However, this work has several limitations derived from the type of sampling used, which does not allow the results to be generalized to the entire population of urban runners. Thus, it is necessary to carry out more work along these lines from races in which runners from additional Spanish cities and international cities take part, with the aim of comparing the groups and the reasons as to why these runners take part in popular races. It is also necessary to include other categories of motives, such as those related to health, that have not been integrated into the scale used in the current study.

## 5. Conclusions

The four following factors were identified that made it possible to identify the motives or reasons why urban runners participate in popular races: personal motives at the psychological and social levels, social motives and interest in running events, the occupation of time and social recognition, and competitive and material motives.

To respond to the objective of the study, three groups of runners were identified with different motives for their participation in popular races: “individual hedonists”, “enthusiasts” and “socializing hedonists”.

Sociodemographic variables related to age, educational level and income level were found to contribute to differentiating the identified groups, as well as variables related to sports habits such as the frequency with which the participants go out to run, their company at the time of going out to run or the level they consider themselves to have as a runner.

“Enthusiastic” runners consider most of the reasons (psychological, physical, social and social recognition) to be important when participating in popular races.

“Individual hedonist” runners are characterized by giving importance to individual psychological and physical motives, while giving little importance to aspects related to social recognition, the occupation of free time, competition or material aspects.

Finally, the group of “socializing hedonists” is characterized by giving importance to not only personal motives but also those of a social nature and an interest in the sport; however, they discard as irrelevant the motives related to external social recognition and those of a competitive or material type.

## Figures and Tables

**Table 1:** Sociodemographic characteristics of the sample.

Variable	Response Option	Mean and Percentage
Age		39.40 (SD ^1^ = 9.38)
Gender	Male	75.5%
	Female	24.5%
Occupation	Employee	76.8%
	Unemployed	10.9%
	Student	6.6%
	Other (retired, pensioner, domestic tasks…)	5.7%
Level of Studies	Primary	5.9%
	Secondary	6.0%
	Baccalaureate/Professional training	30.1%
	University	58.1%
Income Level	Less than 12,000 euros	28.0%
	12,001–18,000 euros per year	22.3%
	18,001–24,000 euros per year	20.4%
	24,001–30,000 euros per year	14.3%
	30,001–36,000 euros per year	6.7%
	More than 36,001 euros per year	8.3%

^1^ SD = Standard Deviation.

**Table 2:** Mean, standard deviation, asymmetry and kurtosis values of the indicators from the scale of motives as to why runners participate in popular races.

Number	Items	Means (SD) ^1^	Asymmetry	Kurtosis
M1	Feeling the pleasure of running	4.01 (0.88)	0.88	−0.05
M2	Fulfilling the goal I set for myself	3.90 (0.91)	0.91	0.12
M3	Checking my physical condition	3.76 (0.84)	0.84	0.32
M4	Competing against my own mark	3.43 (1.08)	1.08	−0.51
M5	Having fun during the competition	4.14 (0.84)	0.84	0.37
M6	Being attracted to the sport	3.93 (0.86)	0.86	−0.10
M7	To meet other runners	3.24 (1.08)	1.08	−0.57
M8	The attractiveness of competitions	3.43 (1.05)	1.05	−0.32
M9	Feeling part of the group of runners	3.22 (1.12)	1.12	−0.69
M10	Being proud of myself	4.12 (.90)	0.90	0.83
M11	Feeling more confident	3.70 (1.12)	1.12	−0.45
M12	To give occupation to my free time	2.64 (1.18)	1.18	−0.83
M13	Making my life more meaningful	2.88 (1.24)	1.24	−0.97
M14	The prestige of this competition	2.33 (1.14)	1.14	−0.63
M15	To demonstrate my interest in the sport	2.77 (1.23)	1.23	−0.93
M16	Others feeling proud of me	2.40 (1.20)	1.20	−0.67
M17	Being a part of my preparation for another sport	2.38 (1.25)	1.25	−0.73
M18	Others seeing me competing with my friends and family	2.08 (1.09)	1.09	0.09
M19	Defeating other colleagues	1.91 (1.05)	1.05	0.36
M20	Wanting to be selected to represent my country	1.40 (0.84)	0.84	5.70
M21	Getting a better time than another teammate	1.80 (1.06)	1.06	0.85
M22	Wanting to get some material stimulus (T-shirt, runner’s bag, etc.)	2.18 (1.21)	1.21	−0.33

^1^ SD = Standard Deviation; 1 = Not Important, 2 = Not Very Important, 3 = Important, 4 = Very Important and 5 = Extremely Important.

**Table 3:** Rotated factorial structure of the scale on the motives as to why runners participate in urban endurance races, including communalities, eigenvalues, and explained variance.

Number	Items	F1	F2	F3	F4	Com. ^1^
	*Factor 1: Psychological and physical motives*					
M2	Fulfilling the goal I set for myself	0.51				0.40
M3	Checking my physical condition	0.33				0.29
M10	Being proud of myself	0.79				0.66
M11	Feeling more confident	0.61				0.58
	*Factor 2: Social motives and interest in running events*					
M1	Feeling the pleasure of running		0.36			0.20
M5	Having fun during the competition		0.52			0.35
M6	Being attracted to the sport		0.54			0.38
M7	To meet other runners		0.83			0.65
M8	The attractiveness of competitions		0.58			0.51
M9	Feeling part of the group of runners		0.63			0.63
	*Factor 3: Occupation of time and social recognition*					
M12	To give occupation to my free time			0.61		0.44
M13	Making my life more meaningful			0.67		0.59
M14	The prestige of this competition			0.71		0.65
M15	To demonstrate my interest in the sport			0.81		0.67
M16	Others feeling proud of me			0.61		0.53
M17	Being a part of my preparation for another sport			0.36		0.32
	*Factor 4: Competitive and material motives*					
M19	Defeating other colleagues				0.90	0.85
M21	Getting a better time than another teammate				0.89	0.77
M22	Wanting to get some material stimulus (T-shirt, runner’s bag, etc.)				0.46	0.30
G H Index		0.80	0.85	0.88	0.91	
Eigenvalue		6.70	1.49	1.17	2.21	
Variance Explained (%)		35.28	7.86	6.14	11.64	
Items		4	6	6	3	

^1^ Com. = Communality.

**Table 4:** Factorial loads, Cronbach’s alpha, composite reliability and average variance extracted values from the scale indicators as to why runners participate in urban endurance races.

Number	Items	λ	α	CR ^1^	AVE ^2^
	*Factor 1: Psychological and physical motives*		0.74	0.81	0.61
M3	Checking my physical condition	0.47			
M10	Being proud of myself	0.79			
M11	Feeling more confident	0.87			
	*Factor 2: Social motives and interest in running events*		0.80	0.80	0.46
M5	Having fun during the competition	0.44			
M6	Being attracted to the sport	0.54			
M7	To meet other runners	0.79			
M8	The attractiveness of competitions	0.68			
M9	Feeling part of the group of runners	0.85			
	*Factor 3: Occupation of time and social recognition*		0.84	0.85	0.49
M12	To give occupation to my free time	0.67			
M13	Making my life more meaningful	0.75			
M14	The prestige of this competition	0.81			
M15	To demonstrate my interest in the sport	0.77			
M16	Others feeling proud of me	0.70			
M17	Being a part of my preparation for another sport	0.45			
	*Factor 4: Competitive and material motives*		0.78	0.82	0.62
M19	Defeating other colleagues	0.94			
M21	Getting a better time than another teammate	0.86			
M22	Wanting to get some material stimulus (T-shirt, runner’s bag, etc.)	0.49			

^1^ CR = Composite Reliability; ^2^ AVE = Average Variance Extracted.

**Table 5:** Correlations between scale factors regarding the motives for runners participating in urban endurance races.

Factors	F1	F2	F3	F4
Factor 1: Psychological and physical motives	0.78			
Factor 2: Social motives and interest in running events	0.56 **	0.68		
Factor 3: Occupation of time and social recognition	0.52 **	0.56 **	0.70	
Factor 4: Competitive and material motives	0.25 **	0.28 **	0.45 **	0.79

** indicates that the correlation is significant at the 0.01 level (bilateral). The diagonal offers the values of the √AVE.

**Table 6:** Average scores for each variable in the three clusters (obtained through the K-averages method).

Number	Items	1 = Individual Hedonists (n = 276)	2 = Enthusiasts (n = 312)	3 = Socializing Hedonists (n = 349)	F	*p* Value
	*Factor 1: Psychological and physical motives*	3.18	4.32	3.99	266.22	<0.001 *
M3	Checking my physical condition	3.36	4.12	3.75	69.59	<0.001 *
M10	Being proud of myself	3.45	4.53	4.30	154.40	<0.001 *
M11	Feeling more confident	2.73	4.31	3.93	238.89	<0.001 *
	*Factor 2: Social motives and interest in running events*	2.91	4.12	3.65	319.70	<0.001 *
M5	Having fun during the competition	3.75	4.37	4.24	47.61	<0.001 *
M6	Being attracted to the sport	3.38	4.31	4.01	108.30	<0.001 *
M7	To meet other runners	2.50	3.90	3.23	164.74	<0.001 *
M8	The attractiveness of competitions	2.71	4.08	3.42	170.76	<0.001 *
M9	Feeling part of the group of runners	2.23	3.93	3.36	270.19	<0.001 *
	*Factor 3: Occupation of time and social recognition*	1.62	3.50	2.48	967.46	<0.001 *
M12	To give occupation to my free time	1.71	3.74	3.05	208.49	<0.001 *
M13	Making my life more meaningful	1.36	3.37	2.16	351.77	<0.001 *
M14	The prestige of this competition	1.71	3.83	2.66	468.57	<0.001 *
M15	To demonstrate my interest in the sport	1.48	3.41	2.23	415.06	<0.001 *
M16	Others feeling proud of me	1.72	3.26	2.13	331.35	<0.001 *
M17	Being a part of my preparation for another sport	1.71	3.74	3.05	165.68	<0.001 *
	*Factor 4: Competitive and material motives*	1.51	2.76	1.61	273.54	<0.001 *
M19	Defeating other colleagues	1.43	2.75	1.53	223.51	<0.001 *
M21	Getting a better time than another teammate	1.41	2.61	1.38	196.52	<0.001 *
M22	Wanting to get some material stimulus (T-shirt, runner’s bag, etc.)	1.68	2.92	1.91	110.20	<0.001 *

* Statistically significant mean differences *p* < 0.001.

**Table 7:** Characteristics of the different groups (clusters).

Variable	Response Option	1 = Individual Hedonists (n = 276)	2 = Enthusiasts (n = 312)	3 = Socializing Hedonists (n = 349)
Age F(2, 934) = 4.07, *p* = 0.02		39.52 (SD ^1^ = 8.80)	38.25 (SD ^1^ = 9.58)	40.32 (SD ^1^ = 9.56)
Gender χ^2^(2) = 4.66, *p* = 0.09	Male	79.35%	75.96%	71.92%
	Female	20.65%	24.04%	28.08%
Occupation Χ^2^(6) = 5.58, *p* = 0.47	Employee	80.80%	73.72%	76.50%
	Unemployed	9.06%	11.54%	11.75%
	Student	5.07%	8.65%	6.02%
	Other (retired, pensioner, domestic tasks…)	5.07%	6.09%	5.73%
Level of studies χ^2^(6) =3 1.31, *p* = <0.001 C^2^ = 0.18	Primary	3.62%	9.29% ^(1) (3)^	4.58%
	Secondary	3.26%	9.29% ^(1)^	5.16%
	Baccalaureate/ Professional training	25.36%	32.69%	31.52%
	University	67.75% ^(2)^	48.72%	58.74% ^(2)^
Income level Χ^2^(10) = 21.73, *p* = 0.02 C^2^ = 0.15	Less than 12,000 euros	24.28%	34.94% ^(1) (2)^	24.64%
	12,001–18,000 euros per year	21.01%	24.04%	21.78%
	18,001–24,000 euros per year	18.84%	17.95%	23.78%
	24,001–30,000 euros per year	17.39%	12.50%	13.47%
	30,001–36,000 euros per year	8.33%	5.13%	6.88%
	More than 36,001 euros per year	10.14%	5.45%	9.46%
How often you run during the week? Χ^2^(6) = 14.79, *p* = 0.02 C^2^ = 0.12	Five or more times a week	6.16%	7.37%	4.30%
	Three to five times a week	48.19%	54.81%	53.87%
	Once or twice a week	34.78%	33.01%	36.68%
	Less frequently	10.87% ^(2) (3)^	4.81%	5.16%
Preferred distance in popular races Χ^2^(10)=13.67, *p* = 0.19	Less than 7.5 km	41.67% ^(3)^	34.29%	31.52%
	Between 7.5 km and 10 km	23.19%	30.45%	31.52%
	Between 10 km and 15 km	20.29%	21.79%	24.36%
	Between 15 km and 20 km	7.97%	9.62%	8.02%
	Between 20 km and 30 km	4.35%	2.24%	2.87%
	More than 30 km	2.54%	1.60%	1.72%
How do you usually run? Χ^2^(2) = 7.33, *p* = 0.03 C^2^ = 0.09	Alone	65.22% ^(2)^	54.49%	61.32%
	Accompanied	34.78%	45.51% ^(1)^	38.68%
Level you consider you have as a runner Χ^2^(4) = 26.31, *p* = <0.001 C^2^ = 0.16	High level	4.35%	5.45%	2.87%
	Intermediate	50.36%	66.67% ^(1) (3)^	52.72%
	Low level	45.29% ^(2)^	27.88%	44.41% ^(2)^
Are you member of a sports club? Χ^2^(2) = 3.92, *p* = 0.14	Yes	37.32%	45.19%	42.98%
	No	62.68%	54.81%	57.02%
Are you sport federated? Χ^2^(2) = 2.10, *p* = 0.35	Yes	6.16%	5.45%	3.72%
	No	93.84%	94.55%	96.28%
Distance usually run weekly F(2934) = 1.82, *p* = 0.16		26.93 (SD ^1^ = 16.71)	29.04 (SD ^1^ = 16.08)	26.97 (SD ^1^ = 14.71)
Years running F(2934) = 0.25, *p* = 0.78		7.35 (SD ^1^ = 7.76)	6.91 (SD ^1^ = 7.85)	7.28 (SD ^1^ = 8.57)
Participation in half marathons F(2934) = 0.25, *p* = 0.68		4.51 (SD ^1^ = 10.79)	5.68 (SD ^1^ = 21.22)	5.11 (SD ^1^ = 14.95)
Participation in marathons F(2934) = 0.25, *p* = 0.87		0.89 (SD ^1^ = 2.84)	0.78 (SD ^1^ = 2.25)	0.84 (SD ^1^ = 2.74)

^1^ SD = Standard Deviation; ^2^ C = Contingency Coefficient; * Indicates statistically significant relationship or statistically significant mean differences * *p* < 0.05; ** *p* ≤ 0 0.01; *** *p* ≤ 0.001; ^(1) (2) (3)^ Results are based on bilateral tests with a level of significance 0.05. The results table shows for each significant pair the key of the group of runners with the proportion of the smallest column below the group of runners with the largest proportion of the column.
